# Using explainable machine learning to uncover the kinase–substrate interaction landscape

**DOI:** 10.1093/bioinformatics/btae033

**Published:** 2024-01-19

**Authors:** Zhongliang Zhou, Wayland Yeung, Saber Soleymani, Nathan Gravel, Mariah Salcedo, Sheng Li, Natarajan Kannan

**Affiliations:** School of Computing, University of Georgia, Athens, GA 30602, United States; Institute of Bioinformatics, University of Georgia, Athens, GA 30602, United States; School of Computing, University of Georgia, Athens, GA 30602, United States; Institute of Bioinformatics, University of Georgia, Athens, GA 30602, United States; Department of Biochemistry and Molecular Biology, University of Georgia, Athens, GA 30602, United States; School of Data Science, University of Virginia, Charlottesville, VA 22903, United States; Institute of Bioinformatics, University of Georgia, Athens, GA 30602, United States; Department of Biochemistry and Molecular Biology, University of Georgia, Athens, GA 30602, United States

## Abstract

**Motivation:**

Phosphorylation, a post-translational modification regulated by protein kinase enzymes, plays an essential role in almost all cellular processes. Understanding how each of the nearly 500 human protein kinases selectively phosphorylates their substrates is a foundational challenge in bioinformatics and cell signaling. Although deep learning models have been a popular means to predict kinase–substrate relationships, existing models often lack interpretability and are trained on datasets skewed toward a subset of well-studied kinases.

**Results:**

Here we leverage recent peptide library datasets generated to determine substrate specificity profiles of 300 serine/threonine kinases to develop an explainable Transformer model for kinase–peptide interaction prediction. The model, trained solely on primary sequences, achieved state-of-the-art performance. Its unique multitask learning paradigm built within the model enables predictions on virtually any kinase–peptide pair, including predictions on 139 kinases not used in peptide library screens. Furthermore, we employed explainable machine learning methods to elucidate the model’s inner workings. Through analysis of learned embeddings at different training stages, we demonstrate that the model employs a unique strategy of substrate prediction considering both substrate motif patterns and kinase evolutionary features. SHapley Additive exPlanation (SHAP) analysis reveals key specificity determining residues in the peptide sequence. Finally, we provide a web interface for predicting kinase–substrate associations for user-defined sequences and a resource for visualizing the learned kinase–substrate associations.

**Availability and implementation:**

All code and data are available at https://github.com/esbgkannan/Phosformer-ST. Web server is available at https://phosformer.netlify.app.

## 1 Introduction

Protein phosphorylation, catalyzed by protein kinase enzymes, is a prevalent post-translational modification (PTM) that underlies nearly all signaling pathways in eukaryotic cells (Manning *et al.* 2002). The human genome encodes over 500 protein kinases that selectively recognize and modify proteins on serine (S), threonine (T), or tyrosine (Y) residues. Although phosphoproteomics studies have identified over 200 000 phosphorylation sites in the human proteome (Hornbeck *et al.* 2012), assigning these modifications to specific kinases remains a significant challenge due to incomplete knowledge of kinase–substrate specificity determinants.

Because of the transient nature of kinase–substrate interactions and the cost and time associated with the experimental characterization of kinase substrates, there has been considerable effort in the development of machine learning models for kinase–substrate prediction (Wang *et al.* 2017, Luo *et al.* 2019, Yang *et al.* 2021, Kirchoff and Gomez 2022, Zhou *et al.* 2023). In general, these models are trained on known phosphosites in databases such as PhosphoSitePlus (Hornbeck *et al.* 2012), then used to predict new kinase–substrate associations. Despite advanced performance, two significant challenges persist in these machine learning-based predictors. The first relates to the composition of the datasets used to train these models. Most often, these models lack negative data (Hornbeck *et al.* 2012), a crucial element for reducing the incidence of false-positive predictions. The second challenge stems from the model’s design and training objectives, with many models confined to making predictions on a restricted set of well-studied kinases (Wang *et al.* 2017, Luo *et al.* 2019, Yang *et al.* 2021, Kirchoff and Gomez 2022, Zhou *et al.* 2023), limiting their application in discovering novel kinase–substrate interactions for understudied kinases. Therefore, creating a kinase–substrate phosphorylation predictor that assimilates both experimentally validated positive and negative data into one framework with the capability of generalizing beyond the seen kinase–substrate pairs could significantly augment the model’s pattern recognition ability and enhance its capacity for zero-shot predictions on new kinases.

Recently, Johnson *et al.* (2023) profiled the substrate specificity of 303 serine/threonine kinases using a combinatorial peptide library approach. The dataset generated from this high-throughput *in vitro* study offers an excellent platform for machine learning as it (i) uniformly samples a diverse array of serine/threonine kinases, and (ii) provides experimentally validated positive (phosphorylated) and negative (not phosphorylated) examples. This allows us to define a focused learning objective of predicting kinase-specific phosphorylation in an *in vitro* setting that can be further refined to predict *in vivo* substrates based on cellular localization or cell type-specific expression.

We propose Phosformer-ST, a multitask transformer model achieving state-of-the-art performance predicting Serine/Threonine (ST) phosphorylation by kinases. Leveraging a comprehensive dataset, our model recognizes a wide range of substrate specificity motifs with high granularity. Through analyzing the model’s learning process and decision-making strategy, we gained insights into its inner workings. The model employs a unique method of first recognizing a phosphorylatable peptide sequence, then ruling out which kinases are not likely to phosphorylate the peptide by a process of elimination. Additionally, our model demonstrates versatility by enabling zero-shot predictions. By leveraging a universal format accepting a 15-residue peptide and kinase domain sequence pair, we showcase its capability to predict phosphorylation outcomes for kinases not used in the peptide library screens.

Learning based on primary sequences alone, we show that our deep learning model has developed a unique understanding of kinase–substrate relationships and a unique strategy for phosphosite prediction, considering both substrate motif patterns and kinase evolutionary and functional relationships. This insight into how machines solve problems can enable end-to-end cycles of designing, building, and learning the substrate specificity determinants of protein kinases through human–computer collaborations.

## 2 Materials and methods

### 2.1 Dataset curation and augmentation

We curated positive and negative examples from a study by Johnson *et al.* (2023) that defined substrate specificity profiles for 303 kinases using *in vitro* peptide arrays, then computationally mapped these profiles to 89 784 experimentally determined serine/threonine phosphosites, which are represented as 15-mer peptides. This provides a total of 27 204 552 unique kinase–peptide combinations. Each combination has a percentile score where scores above 90 are considered positive examples, as defined by Johnson *et al.* (2023).

Out of the 303 kinases, we excluded 3 kinases [BCKDK, PDK1, and PDK4 (UniProt accessions O14874, Q15118, and Q16654)] that are unrelated to the protein kinase fold. We also removed duplicate peptide sequences and peptides that could not be mapped back to their full-length sequences, leaving us with 86 043 phosphosite peptides. Based on the full sequence contexts of these peptides, we also curated 884 203 unique serine/threonine sites with no evidence of phosphorylation. This allows us to define two types of negative examples (Zhou *et al.* 2023).:

- **Hard negatives**: peptides with evidence of being phosphorylated, but not by the paired kinase.
- **Easy negatives**: peptides with no evidence of being phosphorylated by any kinase.

Next, we performed sequence identity-based clustering on the positive and two negative datasets. These cluster assignments will later be used for filtering. Clustering our 300 kinase sequences at 70% identity defined 187 clusters by Cluster Database at High Identity with Tolerance program (CD-HIT). We split our 970 246 curated peptides, based on the central position, for a total of 596 202 serine sites and 364 204 threonine sites. We performed greedy clustering with a cutoff Hamming distance of 5, based on the 11 central residues—the 2 flanking residues on either end were not considered. This resulted in 39 077 clusters of serine sites and 34 470 clusters of threonine sites. CD-HIT was not used for peptide clustering because it performs a pairwise alignment prior to calculating sequence identity that would lose position-specific uniformity of the peptide when clustering.

We built a representative dataset based on our clustering results. We defined 1 010 423 positive examples using all kinase–peptide pairs with percentile scores above 90%, where each pair must have been previously assigned to a unique combination of kinase and peptide clusters. To increase the diversity of our dataset, we randomly selected the representative pair from each unique cluster combination. This strategy allows for the use of multiple members from a given peptide cluster if it is paired with multiple kinases, and vice versa. We defined 3 080 095 hard negative examples using all kinase–peptide pairs with percentile scores below 50%, applying the same filtering strategy. We defined 8 681 574 easy negative examples—which do not have percentile scores. These were non-exhaustively generated by randomly pairing kinases with peptides that have no evidence of phosphorylation and removed similar examples by the same filtering strategy as the hard negative set.

**Figure 1.**
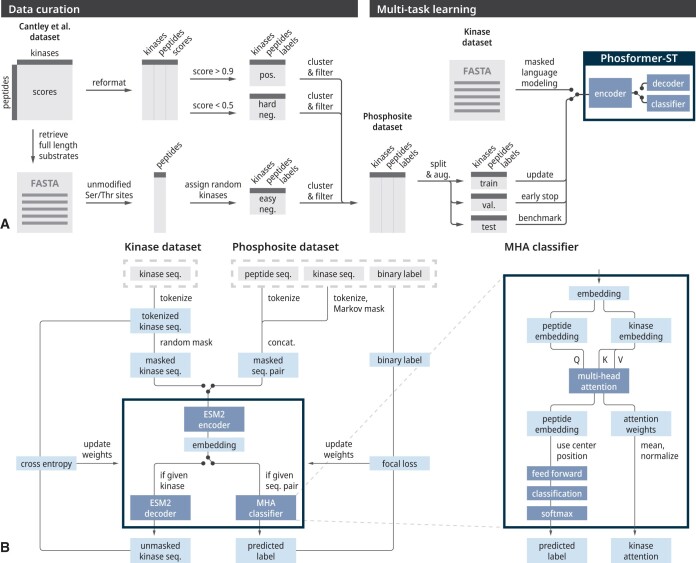
The Phosformer-ST model architecture. (A) A full schematic of our data curation (left) and multitask learning (right) pipelines. (B) The architecture of the Phosformer-ST model classifies kinase–substrate pairs as positive or negative interactions.

Finally, we split positive examples into non-overlapping training, validation, and testing sets at a 60:20:20 ratio. Based on the number of positive examples, we further assigned negative examples such that the ratio of positives to hard negatives to easy negatives was 1:1:1 in the validation and testing sets. The validation dataset was used for hyperparameter tuning while the testing dataset was held out as a standard benchmark dataset for comparing all models (Fig. 1A).

In addition, we employed several data augmentation strategies to prevent overfitting and promote the training of more robust models. These strategies are described in detail in Supplementary Material S1. In brief, a variety of strategies from shifting of domain boundaries, random and Markov masking of sequences, and multi-level negative sampling was employed for data augmentation, the effect of these data augmentation strategies is described in Supplementary Material S3.

### 2.2 Model training

We built Phosformer-ST by fine-tuning pretrained ESM2 protein language models (Rives *et al.* 2021). Pretrained models were previously taught to understand the “language of life” by masked language modeling (MLM) on biologically observed sequences. Our model is trained to predict kinase-specific phosphorylation based on two inputs—a 15-mer peptide sequence and an unaligned kinase domain sequence. Given the source of our training data, predictions should be interpreted in an *in vitro* context.

Prior to being passed into the model, inputs are preprocessed by an Evolutionary Scale Modeling (ESM) tokenizer (Rives *et al.* 2021). A single sequence input—used for MLM—would begin with the <cls> token, followed by the residue tokens, before ending with the <eos> token. As for two sequence inputs, a tokenized peptide–kinase pair begins with the <cls> token, followed by 15 residue tokens corresponding to the 15-mer peptide, then a <eos> token, followed by the kinase sequence residue tokens, before ending with the <eos> token. Consequently, the potential phosphosite is always located on the ninth token.

Phosformer-ST was simultaneously trained on two separate objectives by multitask learning. This was implemented using a shared encoder which outputs an embedding vector that can be passed to either a decoder (for MLM) or a classifier (for kinase-specific phosphosite prediction) (Fig. 1B).

#### 2.2.1 Kinase MLM

In order to learn the distinct features of kinase fold enzymes, the MLM objective was trained on a previously curated dataset of 295 320 diverse kinase domain sequences spanning 18 832 organisms (Zhou *et al.* 2023). We utilized Cross Entropy loss and set the learning rate to start from 2e-5 and decreased linearly to 0 during training.

#### 2.2.2 Kinase-specific phosphosite prediction

Given two sequences denoting a peptide–kinase pair, the model must learn to predict whether the kinase can phosphorylate the peptide *in vitro*. Peptide–kinase embedding pairs from the encoder are separated, then passed through a single-layer multi-head attention block which models the attention of kinase residue embeddings toward peptide residue embeddings. The final prediction is generated from the embedding of the middle residue of the peptide—the potential phosphosite—which is directed toward a binary classification layer. We utilized Cross Entropy loss and set the learning rate to start from 2e-5 while decreasing linearly to 0 during training. Both MLM and classification used AdamW optimizer.

We implemented a customized task manager that chooses between MLM and classification tasks randomly during each training batch. The model was trained for 3 epochs on 8 NVIDIA A6000 GPUs with an effective batch size of 64 per device using gradient accumulation.

### 2.3 Explainable AI for model interpretation

As the models become larger and more complex, biologists may find it challenging to understand its mechanisms and trust its predictions. To address these concerns and delve deeper into our model’s learning process, we have employed various explainable AI methods from both global and local perspectives. At the global level, we examined how the model progressively learns the underlying patterns between different kinase–substrate specificities and their evolutionary relationships. At the local level, we focused on the specific residues and motifs that contribute to the predicted kinase–substrate interactions.

#### 2.3.1 Global interpretations

**Figure 2.**
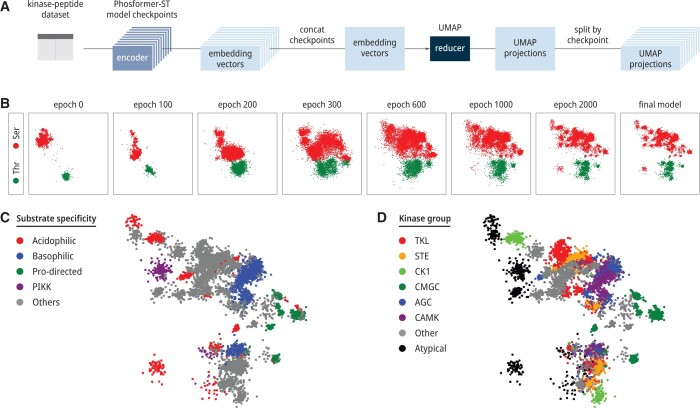
UMAP projections illustrate the model’s learning process. (A) A flow chart depicts our process for quantifying the relationships between embeddings generated from Phosformer-ST at various checkpoints, corresponding to different training epochs. (B) A continuous series of UMAP projections show the relationships between embeddings—from the encoder—at various training epochs, indicated above each plot. (C) The UMAP projections of the final model, re-colored by kinase specificity groups (Johnson *et al.* 2023). (D) The UMAP projections of the final model, re-colored by evolutionary groups (Hanks and Hunter 1995). An interactive scatter plot of the final model with labels for all data points is available in Supplementary File X1.

To generate global interpretations of the learning process from a spatial and temporal perspective (Fig. 2A), we employed Uniform Manifold Approximation and Projection (UMAP) (McInnes *et al.* 2018) for dimensionality reduction. For the spatial perspective, we first inputted the kinase along with its phosphorylated substrates into our trained model. This process yielded intermediate hidden states, denoted as the embedding *E*. The embedding vector has dimensions of N×d, where *N* represents the variable sequence length, and *d* denotes the predefined embedding dimensions. Given that the center of the substrate contains the actual phosphorylation sites and is linked to the classification head during training, we used the embedding of this site to represent the entire kinase–substrate pair. We then fed the embeddings from different kinase–substrate pairs into UMAP to reduce the dimension to 2 and colored them by substrate center amino acid type (Fig. 2B), substrate specificity (Fig. 2C), and kinase evolutionary group (Fig. 2D).

From the temporal perspective, we saved various checkpoints of our model throughout the training. To align representations from different training phases, we concatenated the representations generated by different checkpoints before applying UMAP for dimension reduction. Once the 2D representations were obtained, we separated the projections back into their respective checkpoints. Our workflow is illustrated in Fig. 2A.

#### 2.3.2 Local interpretations

**Figure 3.**
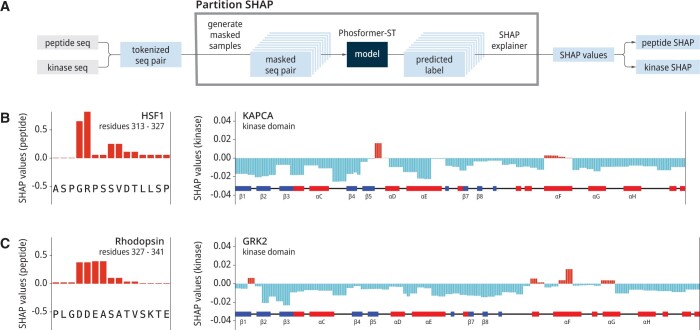
SHAP values for substrate–kinase pairs. (A) A flow chart depicts our process for calculating SHAP values for a given substrate–kinase pair. Bar plots display the SHAP values for two substrate–kinase pairs: (B) HSF1 and KAPCA, and (C) Rhodopsin and GRK2. Across the x-axis, we show the peptide sequences (left) as well as the secondary structure of the kinase (right). SHAP values were estimated using the Partition method.

For local interpretations, we turned to a well-established method in explainable AI known as SHapley Additive exPlanations (SHAP) (Lundberg and Lee 2017), which assigns the contribution of each feature to the prediction for a specific instance. SHAP values explain model predictions starting from an expected value, the model’s average prediction. The SHAP values then show how each feature causes predictions to differ from this average, thereby better illustrating the input-prediction correlations. To calculate these SHAP values, we utilized the partition-based explainer within SHAP. The partition-based method presents two advantages: (i) It is model-agnostic and, when utilizing a balanced partition tree, maintains a quadratic exact runtime (in terms of the number of input features). (ii) It consistently assigns credit to groups of correlated features, mirroring the impact that the set of features would have if treated collectively. Lastly, we separated the final SHAP values into kinase and peptide parts to facilitate easier analysis. The detailed workflow is depicted in Fig. 3A.

### 2.4 Benchmarking

#### 2.4.1 Comparing with other methods

To fully evaluate our model’s performance, we developed multiple levels of benchmarks using different metrics. First, from the kinase-centric perspective, we benchmarked our models using a hold-out testing set of examples that were neither used for training nor early stopping. Specifically, we used two separate testing sets to benchmark different aspects of model performance. To quantify the model’s ability to discern kinase-specific phosphosites, we calculate accuracy, area under the receiver operating characteristic curve (AUC ROC), and area under the precision recall curve (AUC PRC) using a testing set consisting of a 1:1 ratio of positives to hard negatives. To quantify the model’s ability to identify phosphorylatable sites in general, we calculate false positive rate (FPR) from a testing set consisting solely of easy negatives. This evaluation is designed to investigate the model’s ability to accurately identify non-phosphorylatable sites, thereby assessing its potential for false-positive recognitions in a more controlled setting. In addition, we tested our model’s performance from a substrate-centric perspective where we generate predictions for substrates against all kinases by ranking the prediction scores. Subsequently, we calculated Hit@1 accuracy on kinase family and group levels for all models. This evaluation helps prioritize which kinase is primarily responsible for phosphorylating a specific peptide, thus providing an evaluation from a more practical standpoint. Because most compared models only make predictions on a restricted number of groups or families, the group-level score is for kinases within CAMK, CMGC, AGC, and atypical groups and the family-level score is for kinases within PKC, CDK, MAPK, CK2 families. The final Hit@1 score is the macro-average across all groups and families (Supplementary Material S2).

### 2.5 Zero-shot prediction capacity

The unique capability of our model is its ability to predict on any kinase–substrate pairs, irrespective of their presence during training. This attribute originates from two primary sources. First, the ESM backbone encoder, which our model depends on, was pretrained on a comprehensive database of protein sequence data. This pretraining empowers the model to grasp the “language of proteins” effectively, allowing it to perform various downstream tasks. Second, our multitask learning framework further guides the model toward a refined understanding of the “language of kinases” by transferring knowledge between various family members. We constructed a new train-test data partition to validate our hypothesis and separately trained three distinct models.

We departed from the traditional approach of randomly withholding data from kinase–substrate pairs for the dataset. Instead, we randomly chose approximately 10% of kinases and excluded all their associated kinase–substrate pairs. We designated this out-of-distribution testing data as “Test set 2” while randomly dividing a portion from the training dataset and naming it “Test set 1.” Given that the model has not been exposed to such kinases during training, this benchmark will assist us in evaluating the model’s zero-shot capabilities. In total, we trained three diverse models. Although the first model employed the same architecture as the others, its encoder weights were randomly initialized. The second model utilized weights imported from the pretrained ESM model. The final model was trained with the pretrained weights and our multitask objective.

## 3 Results

### 3.1 Performance evaluation and comparison to other models

In our study, Phosformer-ST was benchmarked against five recent phosphosite prediction methods using two distinct test sets. The first test set was employed to determine AUC ROC, AUC PRC, and Hit@k scores with a mixture of positive and “hard negative” examples, while the second set, composed solely of “easy negative” examples, was used to evaluate FPR scores. To meet the input requirements of other methods, additional features like kinase group/family labels and protein–protein interactions had to be included, as Phosformer-ST only utilizes the kinase domain and peptide sequences. Several of these methods employ multiple family/group-specific models, thus reducing their universality. However, Phosformer-ST can theoretically process inputs from any kinase. Phosformer-ST outperforms other models in both prediction accuracy as well as maintaining comparably low false predictions (Table 1). Detailed performance benchmarks for each kinase family are available in Supplementary File X3.

**Table 1.** Comparison of our model with several most recently published kinase-specific phosphorylation predictors including MusiteDeep (Wang *et al.* 2017), DeepPhos (Luo *et al.* 2019), PhosIDN (Yang *et al.* 2021), and EMBER (Kirchoff and Gomez 2022).^a^

Model	AUC ROC	AUC PRC	FPR	Hit@1 Group	Hit@1 Family
MusiteDeep	0.971	0.974	0.057		0.776
DeepPhos	0.8049	0.769	0.152	0.435	0.761
PhosIDNSeq	0.811	0.766	0.131	0.417	0.690
PhosIDN	0.822	0.799	0.005	0.461	0.712
EMBER	0.729	0.250	0.094		0.263
Phosformer	0.581	0.592	0.065	0.520	0.794
Ours	**0.992**	**0.992**	**0.019**	**0.683**	**0.822**

a Note that MusiteDeep and EMBER only allows family-level predictions. The performance of Phosformer-ST ("Ours") is highlighted in bold.

### 3.2 Performance evaluation for zero-shot prediction

While most kinase-specific phosphorylation prediction models focus on a limited set of kinase–substrate pairs (Wang *et al.* 2017, Luo *et al.* 2019, Yang *et al.* 2021), the unique advantage of our model is its ability to make predictions beyond the seen kinase–substrate pairs. This capability arises from our multitask training scheme, in which the model simultaneously learns to predict kinase–substrate pairs and comprehend the broader kinase universe. To test this, we conducted a specialized evaluation in which a subset of kinases was withheld from the training phase to see how the model performs in such scenarios. We developed three models: the first uses the ESM architecture with randomized weights which lacks the initial pretraining; the second employs the ESM pretrained weights but without multitask learning; and the final model uses both the pretrained ESM and multitask learning. The results from Table 2 suggest that ESM pertaining enhances generalizability to the unseen kinase dataset compared to the randomized model. However, the best performance was achieved by our final model, which integrates both ESM pretrained weights and multitask training, significantly outperforming the other two models.

**Table 2.** Results of an ablation experiment.^a^

Model	AUC ROC seen kinase	AUC PRC seen kinase	AUC PRC unseen kinase	AUC ROC unseen kinase
Random model	0.985	0.985	0.829	0.833
w/o multitask	0.978	0.979	0.860	0.863
w/multitask	**0.995**	**0.995**	**0.885**	**0.886**

a Specifically, the mask language modeling part was removed, or the ESM encoder was randomized and a select list of kinase–substrate pairs were held out in the training to benchmark the zero-shot performance of the model.

### 3.3 Phosformer-ST learns multiple layers of underlying patterns for kinase–substrate pairs via multitask fine-tuning

We investigate the learning process, i.e. how the model develops a strategy for kinase-specific phosphosite prediction. As described in the methods, the training process began from a pretrained ESM2 protein language model, which generally understands biologically observed protein sequences (Rives *et al.* 2021). Leveraging this prior knowledge, the model was further trained to predict kinase-specific phosphosites.

We randomly sampled 30 true positive examples from each kinase (9000 total), then investigated how the model interpreted this sample dataset of peptide–kinase pairs at various training epochs. The model’s interpretation of a given pair is represented by sequence embedding. To establish a common frame of reference for comparison, we specifically analyze the phosphosite residue embedding. We visualize the relationships between embeddings from different training epochs using UMAP projections (Fig. 2A).

Before being trained for phosphosite prediction, the pretrained ESM2 model (epoch 0) distinguishes between Ser/Thr sites. The additional variability of each cluster reflects the Transformer model’s innate ability to encode the sequence context. Early in training, the model quickly merges Ser/Thr sites into a single supercluster (epoch 300). For the remainder of the training, the model slowly breaks up the supercluster into many smaller distinct clusters, which reflect a complex organization based on substrate specificity motifs and evolutionarily related kinases families (Fig. 2B and C). We hypothesize that this unique pattern originates from the multitask training scheme. The classification objective encourages the model to capture more details about substrate specificity. In contrast, masked language modeling ensures the model grasps the underlying evolutionary similarity between different kinase families. This behavior dramatically improves the model’s capacity for recognizing unseen kinase–substrate pairs, thus enabling zero-shot predictions.

The visualization of learned embedding clusters, examined across various training steps, offers a fresh perspective to better understand how the model incrementally reorganizes existing knowledge and acquires new domain-specific knowledge during fine-tuning. This method can be broadly applied to probe the learning process of deep learning models.

### 3.4 Phosformer-ST develops a unique strategy for phosphosite prediction

We further investigate how the model utilizes peptide–kinase sequence information in decision-making. To do this, we use SHAP, which allows us to estimate the contribution of each residue toward the final prediction (Štrumbelj and Kononenko 2014). Contributions can be either positive or negative, where the final prediction is the sum of all contributions. Residues with positive contributions support a positive prediction; residues with negative contributions support a negative prediction.

We analyze SHAP values for two well-studied substrate–kinase pairs (Fig. 3). Overall, the peptide sequence information contributes toward positive decisions, while the kinase sequence information contributes toward negative choices. This general trend is observed across the entire dataset (Supplementary File X5). The highest positive contributions correspond to known substrate specificity determinants in our examples. For instance, when given a known substrate peptide–kinase for cAMP-dependent protein kinase PKA (KAPCA), a basophilic kinase (Zhu *et al.* 2005), SHAP assigns the highest positive contribution toward the R at the substrate’s -3 position (Fig. 3A). In our other example utilizing GRK2, an acidophilic kinase (Pinna and Ruzzene 1996), SHAP assigns the highest positive contributions to the substrate’s acidic residues D and E (Fig. 3C) at the P-4, P-3, and P-2 positions.

Phosformer-ST is capable of identifying substrate specificity motifs. However, the predominantly negative contributions from the kinase sequence information suggest that Phosformer-ST may be using sequence motifs to rule out kinases that cannot phosphorylate the peptide. Overall, our model has developed a unique strategy for kinase-specific phosphosite prediction where, upon recognizing a potentially phosphorylatable peptide motif, the model checks if the peptide was matched with a compatible kinase using the process of elimination, using discriminating sequence motifs as a means for ruling out incompatible kinases.

### 3.5 Phosformer-ST associates each kinase to multiple compatible motifs

In addition to investigating our model’s decision-making strategy, we use SHAP values to analyze how Phosformer-ST perceives its inputs. The SHAP values for a given peptide–kinase pair are a suitable vector representation of how the model perceives the information in decision-making.

We calculated SHAP values for all true positives in our testing set—115 552 kinase–peptide pairs. Upon separating by individual kinases, we cluster SHAP values for corresponding examples using affinity propagation, then plot sequence logos for each cluster. We reiterate that these clusters are not based on substrate sequence but instead on SHAP values, each associated with a substrate sequence.

**Figure 4.**
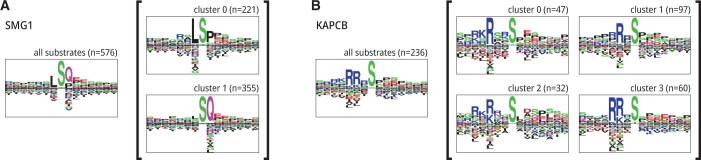
Motif decomposition analysis for two kinases (A) SMG1 and (B) KAPCB. For each kinase, the sequence logos on the left, outside the brackets, show all true positive substrates in the testing set, while the sequence logos in brackets subdivide substrates into clusters. The examples within each cluster share similar SHAP values, indicating that the model interprets them similarly. Sequence logo clustering for all 300 kinases is available in Supplementary File X2. Full clustering results with logos, SHAP values, and attention maps for all 300 kinases are available in Supplementary File X4.

Although the substrate specificity of a given kinase is often represented as a single sequence logo, our results suggest that our model has a unique understanding of substrate specificity where each kinase is associated with multiple highly specific motifs rather than a single general motif (Fig. 4). These results are also supported by our previous analysis of the embedding vectors, which indicated that the fully trained model still separates serine sites from threonine sites (Fig. 2A) while also being able to recognize that a single kinase can be simultaneously capable of phosphorylating both. By describing the specificity of each kinase as a collection of separate specificity motifs, our results provide a means for prioritizing new substrates for future substrate specificity studies. These results indicate that Phosformer-ST has developed a unique understanding of substrate–kinase relationships.

### 3.6 Leveraging Phosformer-ST for zero-shot kinase–substrate interaction prediction

Applying our model to 139 kinases not used in the peptide library screens by Johnson *et al.* (2023), we identified 1 064 168 potential kinase–substrate interactions (Supplementary File X6) using a confidence threshold of 0.5. Peptide profiles of these 139 kinases are shown in Supplementary File X7. In general, the substrate specificity profiles of these kinases closely match the profiles of families to which these kinases belong. However, distinct motif preferences are predicted for subfamilies within some kinase families. For example, Phosformer-ST predicts the substrate profiles of Unc50-like kinase 3 (ULK3) to be more similar to Serine/Threonine Kinase 36 (STK36) [both are not included in the Johnson *et al.* (2023) dataset] than to ULK1 or ULK2. ULK3 and STK36 prefer Arg at the P-3 position, while ULK1/2 prefer Leu at the P-3 position (Supplementary File X8). Lending to the validity of our predictions, previous research has demonstrated that ULK3 and STK36 coordinate in parallel in response to Hedgehog (Hh) signaling stimulus to phosphorylate the latent transcription factor Gli2 at S230/S232 resulting in its conversion from a repressor form into an activator form (Han *et al.* 2019). Of note, in both human and mouse Gli2, there is a conserved Arg found at position P-3 downstream of the phosphorylation site confirming Phosformer-ST’s zero-shot prediction of ULK3 and STK36 favoring Arg at the P-3 position. This further demonstrates the ability of our model to predict substrate specificity of kinases not seen during training and demonstrates its generalizability and potential applications in annotating kinase–substrate phosphorylation atlas across model organisms.

## 4 Discussion

In this study, we present a novel kinase–substrate phosphorylation prediction model that offers several innovative contributions compared with previous works. Firstly, our model includes a comprehensive preprocessing protocol for harnessing large peptide array datasets, in contrast to previous methods, which lacked experimentally validated negative datasets (Wang *et al.* 2017, Luo *et al.* 2019, Yang *et al.* 2021, Kirchoff and Gomez 2022). Secondly, we advanced the field by developing multiple protein-specific data augmentation techniques. These strategies enhance the model’s robustness by mitigating its over-reliance on local contextual information. Thirdly, our model incorporates a multitask learning protocol for protein language models, enabling a balance between acquiring phosphorylation-specific knowledge and comprehending the broader landscape of the kinome. A significant strength of our model is its provision of extensive explainability analysis. Plotting embeddings across training epochs, utilizing SHAP for residue-level explanations, and scrutinizing kinase attention, we facilitate a deeper understanding of the model’s predictions and inherent mechanisms. Our model’s potential for zero-shot learning also demonstrates its utility for predicting kinase–substrate relationships in scenarios with limited or no prior information, further bolstering its practical applicability. To ensure the model’s accessibility to researchers, we have designed a user-friendly web server (Supplementary Material S4), encouraging its widespread use and adoption in kinase research.

While our model represents a significant advancement in the field, we acknowledge several areas for future exploration. Firstly, we recognize that phosphorylation is an inherent biological process not solely regulated by kinases but is also highly dependent on the specific tissue and cellular environment. Thus, a model considering these factors and predicting kinase–substrate interactions in a tissue or cellular environment-specific manner could yield more physiologically relevant predictions. Secondly, we’ve limited the input sequence to the kinase domain region, and the 15-mer substrate, which introduces a strong assumption that the kinase domain is the sole region interacting with the substrate sequence and the 15-mer substrate sequence is the only part interacting with the kinase. Expanding the model and datasets to consider entire kinase and substrate sequences could further capture these interactions, leading to more nuanced and comprehensive predictions.

## Supplementary Material

btae033_Supplementary_Data

## Data Availability

The supplementary data are available at https://zenodo.org/record/8150738.
