# Supplementary material for: Using explainable machine learning to uncover the kinase–substrate interaction landscape
Source: Bioinformatics. 2024 Jan 19;40(2):btae033. doi: 10.1093/bioinformatics/btae033 (PMC10868336; doi:10.1093/bioinformatics/btae033)
Supplement: btae033_Supplementary_Data [file btae033_supplementary_data.zip › Supplemental_legend.pdf]

SUPPLEMENTAL Files

Supplemental File X1. An interactive plot of UMAP projections illustrates the final model with labels for all data points. This plot allows the user to hover and select kinase-substrate pairs and download the selected cluster.

Supplemental File X2. Motif decomposition analysis for all 300 training kinases. For each kinase, the sequence logos on the left, outside the brackets, show all true positive substrates in the testing set, while the sequence logos in brackets subdivide substrates into clusters based on SHAP values.

Supplemental File X3. Detailed performance benchmarks on each kinase individual, family, and group level on three testing datasets (the positive-negative dataset, the shifted positive-negative dataset, and the all-negative dataset). The performance is shown by the accuracy score, PRC-AUC score, AUC-ROC score, and FPR score.

Supplemental File X4. Full clustering results with logos, SHAP values, and attention maps for all 300 kinases.

Supplemental File X5. The SHAP values distribution histogram for all 300 kinases. Overall, the peptide sequence information contributes towards positive decisions, while the kinase sequence information contributes towards negative choices.

Supplemental File X6. Zero-shot prediction for 139 out-of-distribution kinases against 89,784 phosphorylable substrates with a score between 1 and 0. Each column corresponds to a kinase and each row corresponds to a substrate with Uniprot ID, position, and peptide.

Supplemental File X7. Substrate specificity profile weblogo for 139 kinases not included in the experimental peptide array dataset. Phosformer-ST was run for each kinase against the 89,784 phosphorylable substrates and predictions with a score greater than 0.5 was considered positive and used for weblogo generation.

Supplemental File X8. Peptide profile comparison for ULK1, ULK2, ULK3 ULK4, and STK36 kinases. Our model predicts the substrate profiles of ULK3 to be more similar to STK36 than to ULK1 or ULK2. ULK3 and STK36 prefer Arg (R) at the P-3 position, while ULK1/2 prefer Leu (L) at the P-3 position. ULK1 and ULK2 were used in the training of Phosformer-ST while ULK3 ULK4, and STK36 did not have any data included in the training set.
