# Supplementary material for: Using explainable machine learning to uncover the kinase–substrate interaction landscape
Source: Bioinformatics. 2024 Jan 19;40(2):btae033. doi: 10.1093/bioinformatics/btae033 (PMC10868336; doi:10.1093/bioinformatics/btae033)
Supplement: btae033_Supplementary_Data [file btae033_supplementary_data.zip › Supplemental_Material.docx]

Supplementary Material:

**Explainable machine learning uncovers the kinase-substrate interaction landscape**

Zhongliang Zhou^1, †^, Wayland Yeung^2, †^, Saber Soleymani ^1^, Nathan Gravel^2^, Mariah Salcedo^2^, Sheng Li^4,*^ and Natarajan Kannan^2, *^

1 School of Computing, University of Georgia, GA 30605, USA

2 Institute of Bioinformatics, University of Georgia, GA 30605, USA

3 Department of Biochemistry and Molecular Biology, University of Georgia, GA 30605, USA

4 School of Data Science, University of Virginia, VA 22903, USA

^†^ These authors contributed equally to the work,

^*^ To whom correspondence should be addressed

**S1.** Data augmentation method.

Data augmentation techniques help avoid overfitting and promote the training of more robust models by increasing the size of training set via meaningful modifications to the original data. Here, we developed three augmentation strategies that are compatible with our protein sequence inputs.

**Shifting domain boundaries**: Our model encodes the identity of a given kinase based on its unaligned kinase domain sequence. Consequently, the same kinase could be represented by slightly different sequences depending on the defined domain boundaries. In order to make the model robust to this variation, we randomly shift both the N- and C-terminal boundaries of the kinase domain by up to 30 residues. Shifts are performed independently at each terminus. This augmentation also prevents the model from learning arbitrary domain boundaries as a means for identifying the kinase.


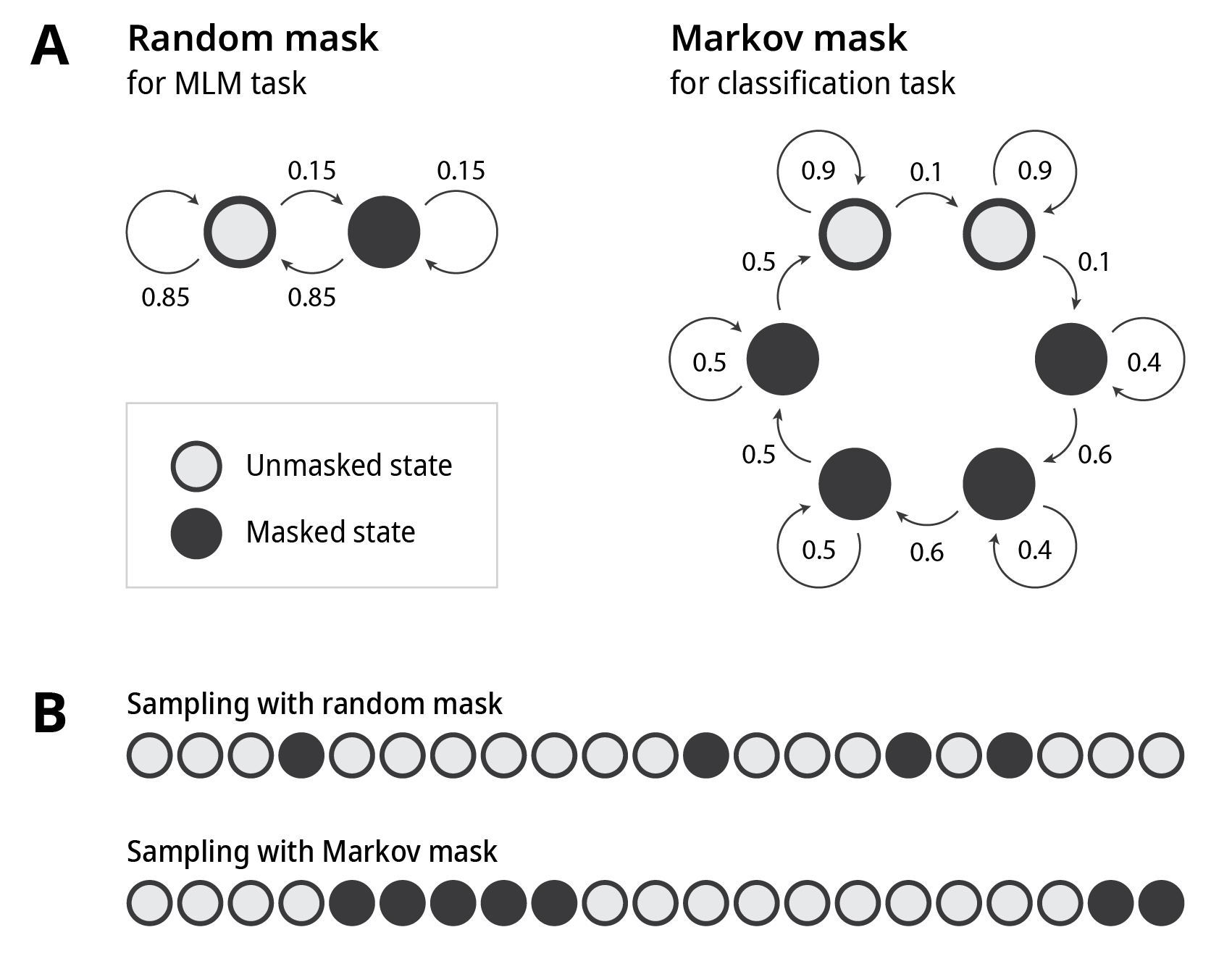


**Kinase sequence masking**: The masking augmentation randomly replaces kinase residue tokens with the <mask> special token to represent the uncertainty of the amino acid. We define two strategies for masking. For MLM, each residue in the kinase sequence is independently given a 15% probability of being replaced with a mask token. This strategy tends to mask tokens evenly across sequences. For kinase-specific phosphosite prediction, we simulate the mask state of each residue as a Markov process, where each residue has a ~ 27% steady-state probability of being masked. We designed a Markov model which yields contiguous spans of masked positions. Overall, these masking augmentations disallow the model from over-relying on a small subset of highly informative neighboring residue positions and encourage the model to consider the entire kinase sequence.

**Multi-level negative sampling**: We previously developed a strategy that leverages different types of negative examples for training, as explained in Section 2.1 of the main paper. In particular, we distinguish between 'easy' and 'hard' negative examples. Through multi-level negative sampling, we sample '*n*' easy negatives and '*m*' hard negatives for each positive example. We represent the overall ratio of positive to negative examples as 1:(*n*+*m*), where '*n*' and '*m*' are tunable parameters that range between 1 and 8.

**S2.** Evaluation metrics.

$$\text{TPR}=\frac{TP}{TP+FN}$$

$$\text{FPR}=\frac{FP}{FP+TN}$$

$$\text{AUC ROC}\approx\sum_{i=1}^{n} \frac{\left( FPR_{i}-FPR_{i-1} \right)\times\left( TPR_{i}+TPR_{i-1} \right)}{2}$$

$$\text{Precision}=\frac{TP}{TP+FP}$$

$$\text{Recall}=\frac{TP}{TP+FN}$$

$$\text{AUC-PRC}\approx\sum_{i=1}^{n} \text{Precision}_{i}\times\left( \text{Recall}_{i}-\text{Recall}_{i-1} \right)$$

$$\text{FPR}=\frac{FP}{FP+TN}$$

$$\text{Macro-Averaged Top-K Accuracy}=\frac{1}{C}\sum_{j=1}^{C} \left( \frac{1}{\left| N_{j} \right|}\sum_{i=1}^{\left| N_{j} \right|} \mathbb{1}\left( y_{ij}\in\text{Top-K}\left( f\left( x_{ij} \right) \right) \right) \right)$$

We mainly rely on the AUC ROC, AUC PRC, FPR and Top@k score to evaluate our model. TP denotes true positive, FP denotes false positive, TN denotes true negative, and FN denotes false negative. The macro-averaged Top-K accuracy score by classes is a measure used to evaluate classification models where the interest is in the average performance across classes, giving the same weight to each class regardless of its frequency. Macro-averaging computes the Top-K accuracy for each class separately and then takes the average. This treats all classes equally, emphasizing the performance on rare classes.

**S3.** Evaluation of data augmentation strategy.

| **model parameters** | **AUC ROC**  Pos+HardNeg  w/o. Shift | **AUC PRC**  Pos+HardNeg  w/o. Shift | **FPR**  EasyNeg  w/o. Shift | **AUC ROC**  Pos+HardNeg  w. Shift | **AUC PRC**  Pos+HardNeg  w. Shift | **FPR**  EasyNeg  w. Shift |
| --- | --- | --- | --- | --- | --- | --- |
| multi-task, (1+0)  no shift, no mask | 0.9896 | 0.9897 | 0.3205 | 0.9166 | 0.9202 | 0.3802 |
| multi-task, (1+1),  no shift, no mask | 0.9812 | 0.9807 | 0.1221 | 0.8497 | 0.8357 | 0.2155 |
| multi-task (1+1),  shift, no mask | 0.9678 | 0.9670 | 0.1203 | 0.9718 | 0.9712 | 0.1231 |
| multi-task (1+1),  no shift, mask | 0.9692 | 0.9687 | 0.1259 | 0.8817 | 0.8850 | 0.1612 |
| multi-task (4+4),  shift, markov | 0.9074 | 0.9113 | 0.0141 | 0.9140 | 0.9182 | 0.0150 |
| multi-task (8+8),  shift, markov | 0.9920 | 0.9919 | 0.0187 | 0.9942 | 0.9940 | 0.0209 |

We systematically evaluate the model using different data augmentation strategies. Specifically, we test: 1. the varying ratios of training in the negative dataset (easy negative versus hard negative), 2. whether to add a shift to the kinase sequence, and 3. whether to apply a mask to the kinase sequence. We performed comparisons on two datasets: 1. The original test datasets containing both positive and hard negative samples, as well as an easy negative dataset, and 2. The augmented test datasets, which involved applying random shifts to the original test datasets. The results suggest that the combination of all data augmentation strategies yields the best-performing model.

**S4.** Web server interface description.

**Prediction Page**: This page enables users to use our dedicated GPU resources and run the model to generate predictions based on their data. Upon inputting substrate and kinase sequences, the model predicts a score between 0 and 1, denoting the likelihood of interaction between a given kinase-peptide pair. This page also allows users to input one or multiple substrates and select a kinase from a drop-down list. Alternatively, users can manually key in the amino acid sequences of a custom protein kinase and peptide sequence.

**UMAP Clustering Page**: This feature extends from the global explanation derived from our final model described in section 3.2. We have constructed an interactive map via the Bokeh package to portray the global distribution of learned kinase-substrate interactions visually. Users can hover over, zoom into, or select any map segment, facilitating an intuitive exploration of the internal relationship between various kinase families and associated substrate specificities.

**Kinase-Substrate Specificity Weblogo Page**: Built based on the kinome tree from Coral this page employs our model's predictions to investigate diverse kinase-substrate specificities using web logo. Users can select any kinase protein, subfamily, or family to view the corresponding web logos for peptide substrates. For each kinase, the interface presents one global web logo for all positive predictions and weblogs for the subclusters, delineated in section 3.4.
